# Network Pharmacology and *In Vivo* Experimental Verification of the Mechanism of the Qing'e Pill for Treating Intervertebral Disc Degeneration

**DOI:** 10.2174/0115734099356426241119051916

**Published:** 2024-12-02

**Authors:** Hui Jin, Huaiyu Ma, Jie Wu, Ruizhe Wu, Haoran Xu, Weixing Chen, Linghui Li, Jingqi Zeng, Fan Wang

**Affiliations:** 1 Department of Orthopedics, the Second Affiliated Hospital of Hunan University of Chinese Medicine, Changsha, 410005, China;; 2 Graduate School, Hunan University of Chinese Medicine, Changsha, 410208, China;; 3 Department of Sports Medicine, Wangjing Hospital of China Academy of Chinese Medical Sciences, Beijing, 100102, China

**Keywords:** Intervertebral disc degeneration, Qing’e Pill, *in vivo* experiments, molecular mechanisms, molecular docking, network pharmacology

## Abstract

**Objective:**

The Qing’e Pill (QEP) is widely used to alleviate low back pain and sciatica caused by Intervertebral Disc Degeneration (IDD). However, its active components, key targets, and molecular mechanisms are not fully understood. The aim of this study is to elucidate the molecular mechanisms through which the QEP improves IDD using database mining techniques.

**Methods:**

Active components and candidate targets of the QEP were identified using the Traditional Chinese Medicine Systems Pharmacology Database and Analysis Platform and the Bioinformatics Analysis Tool for Molecular Mechanisms of Traditional Chinese Medicine. IDD-related targets were obtained from the GeneCards database, and liver- and kidney-specific genes were retrieved from the BioGPS database. The intersection of these candidate targets was analyzed to identify potential targets for the QEP in IDD. A protein-protein interaction network analysis was performed using STRING and Cytoscape 3.7.2 software. Core targets were further analyzed through Gene Ontology (GO) and Kyoto Encyclopedia of Genes and Genomes (KEGG) enrichment analyses. Molecular docking was used to assess the binding affinity of active components to candidate targets, and animal experiments were conducted for validation.

**Results:**

We identified 65 potentially active components of the QEP that corresponded to 1,093 candidate targets, 2,108 IDD-related targets, and 1,113 liver- and kidney-specific genes. Key components included quercetin, berberine, isorhamnetin, and emodin. The primary candidate targets were Wnt5A, CTNNB1, IL-1β, MAPK14, MMP9, and MMP3. The GO and KEGG analyses revealed the involvement of these targets in Wnt signaling, TNF signaling, Wnt receptor activation, Frizzled binding, and Wnt-protein interactions. Molecular docking showed strong binding between these components and their targets. Animal experiments demonstrated that the QEP treatment significantly reduced the expression of Wnt5A, CTNNB1, IL-1β, MAPK14, MMP9, and MMP3 at high, medium, and low doses compared with the model group.

**Conclusion:**

The QEP alleviated IDD by modulating the Wnt/MAPK/MMP signaling pathways and reducing the release and activation of key factors.

## 1 INTRODUCTION

Intervertebral disc degeneration (IDD) can induce chronic lumbar, back, and lower limb pain, along with sensory disturbances, numbness, and functional impairment, significantly reducing patients' quality of life and causing considerable physical and psychological suffering [1]. IDD-induced lower back pain is a pervasive global health issue, with estimates suggesting that approximately 84% of adults will experience this pain at some point in their lives [2]. Consequently, lumbar and back pain significantly contributes to disability and workforce attrition, imposing a considerable socioeconomic burden [3]. The Qing’e Pill (QEP), a traditional Chinese medicine (TCM) formulation that contains Eucommia ulmoides, Psoralea corylifolia, walnut kernels, and garlic, has been shown to alleviate lower back pain caused by IDD. This therapeutic effect is thought to stem from the influence of the QEP on liver- and kidney-related mechanisms, thereby improving patients' quality of life [4, 5]. Despite these findings, the molecular mechanisms and precise targets of the QEP still require further exploration and elucidation.

In recent years, with the continuous advancement of network pharmacology, there have been increasing expectations for its potential to enhance mechanistic research into TCM formulations [6] significantly. As a burgeoning field at the intersection of artificial intelligence and big data, network pharmacology systematically studies drugs by constructing networks that link pharmacologically active compounds with protein targets using various bioinformatics databases. This facilitates an initial understanding of the underlying mechanisms by which TCM, with its multi-constituent and multi-target approach, treats diseases [7]. Additionally, molecular docking is a computational strategy used to predict drug molecule binding modes and affinities with target proteins, and it enhances drug design and optimization processes and helps researchers understand drug interactions with biomolecules [8]. However, the validity of this methodology is somewhat limited due to a lack of experimental validation and data corroboration.

In this study, we examine the interactions between the active components of the QEP and their corresponding targets based on a network pharmacology analysis supported by molecular docking techniques. We explore potential mechanisms by which the QEP may ameliorate IDD and conduct preliminary validation in animal models, providing experimental evidence to support the use of the QEP for treating IDD.

## 2 MATERIALS AND METHODS

### 2.1 Software and Databases

The software and databases used in this study are listed in Table **1**.

### 2.2 Acquisition of Potential Active Ingredients and Targets of the QEP

The chemical constituents of four TCMs, Eucommia ulmoides, walnut kernel, Psoralea corylifolia, and garlic, were acquired from the Traditional Chinese Medicine Systems Pharmacology Database and Analysis Platform (TCMSP) and the Bioinformatics Analysis Tool for Molecular Mechanisms of Traditional Chinese Medicine (BATMAN-TCM) [9, 10]. These databases necessitated an oral bioavailability of at least 30% and a drug-likeness score of no less than 0.18 for inclusion [11]. Moreover, the BATMAN-TCM database specified a score of 20 or higher and a *P*-value of 0.05 or lower. Upon meeting these criteria, the potential active ingredients of the QEP were identified. The target proteins of these potential active components were standardized using the UniProt protein database to obtain target information [12]. These data, along with the potential active components, were imported into Cytoscape 3.9.1 software to create a drug-component-target network, filtering out irrelevant active components and target data. Subsequently, a network topology analysis was performed, and the potential active ingredients of the QEP were prioritized based on their network degree value [13].

### 2.3 Acquisition of Targets Related to IDD and Liver/Kidney Organ Targets

Relevant genes associated with IDD were identified and extracted from the GeneCards database [14] using “Intervertebral Disc Degeneration” as a search keyword. Genes specifically expressed in the liver and kidneys were also identified in the BioGPS database using “liver” and “kidney” as search terms [15].

### 2.4 Construction of the Protein-protein Interaction Network

To identify shared targets among the QEP formula, IDD, and liver/kidney organ systems, an intersecting target set named the “QEP-Liver/Kidney-IDD” was generated. This set was subsequently uploaded to the STRING database using the “multiple proteins” feature, selecting “Homo sapiens” as the species [16]. The STRING database categorizes protein-protein interaction (PPI) confidence levels into four grades: highest confidence at 0.9, high confidence at 0.7, medium confidence at 0.4, and low confidence at 0.15. We selected targets with a high-confidence interaction score of 0.9 and excluded free nodes to construct the PPI network of intersecting targets among the QEP, IDD, and liver/kidney. The results were saved in the tab-separated values file format and imported into Cytoscape 3.7.2 software for visual analysis of the core targets [17].

### 2.5 Validation Set Verification

To validate the differential expression of the “Qing’e Pill-Liver/Kidney-IDD” intersecting targets between healthy individuals and IDD patients, we used the GSE124272 dataset from the GEO database [18]. The dataset included blood samples from eight IDD patients and eight healthy volunteers. These samples were analyzed using the Agilent SurePrint G3 Human Gene Expression Microarray 8x60 K platform to identify differentially expressed genes (DEGs) in the whole blood of IDD patients. Selection criteria required an absolute log2 fold change greater than one and an adjusted P-value below 0.05, ensuring significant differences for the intersecting genes. A differential expression analysis of the GSE124272 dataset was conducted using the limma R package. Additionally, violin plots of the intersecting targets were generated using the ggplot2 and pheatmap packages to provide a comprehensive view of the distribution and trends of the DEGs [19].

### 2.6 Gene Ontology and Kyoto Encyclopedia of Genes and Genomes Enrichment Analyses

The overlapping targets of the QEP, IDD, and liver/kidney organs were uploaded to the Database for Annotation, Visualization, and Integrated Discovery (DAVID) database [20]. Then, the “Functional Annotation” feature was selected, and the Gene Ontology (GO) functional enrichment and Kyoto Encyclopedia of Genes and Genomes (KEGG) pathway analyses were conducted using *Homo sapiens* as the filter criterion with a significance level of *P* < 0.05. The Weishengxin platform was used to visualize the results, and bar and bubble charts were then generated [21].

### 2.7 Molecular Docking Verification

The study initially involved the molecular docking of potential active ingredients and targets in the “TCMs-Active Component-Gene Target” network of the QEP. Initially, Protein Data Bank (PDB) files of pivotal target proteins were downloaded from the PDB [22]. These proteins were processed for dehydration, hydrogen addition, and charge calculation using AutoDockTools 1.5.6 and subsequently saved in PDBQT (auto dock structure) format to serve as docking receptors. Concurrently, the potential active component Mol2 files sourced from the TCMSP database were converted to PDB files using OpenBabel 3.1.1 and then prepared using AutoDockTools 1.5.6, and this involved assigning atomic charges, defining rotatable bonds, and formatting them as PDBQT for docking ligands [23]. Molecular docking was conducted *via* AutoDock Vina 1.1.2 to determine the binding energy values, and the most stable complexes were visualized using PyMOL 2.4.0 [24]. A negative binding energy suggested a viable interaction between the potential active component and the target protein, with more negative values indicating a stronger and more stable interaction. Binding energy values below -5 kcal/mol confirmed a substantial binding affinity between the potential active components and their corresponding target s [25].

### 2.8 *In vivo* Experimental Verification

#### 2.8.1 Preparation of Experimental Animals and Pharmaceuticals

In this study, we used 42 eight-week-old male specific pathogen-free (SPF)-grade Sprague Dawley (SD) rats, each weighing approximately 200 ± 20 g. All rats were provided by Hunan Slake Jingda Experimental Animal Co., Ltd. The company operates under animal production license number SCXK (Xiang) 2019-0009. The research was conducted at the Animal Experiment Center of the Hunan University of Chinese Medicine. This center maintains optimal environmental conditions with well-regulated ventilation, temperatures ranging from 20 to 25°C, humidity levels between 40% and 50%, and a 12 h light/dark cycle. The rats had free access to food and water. We received ethical approval from the Ethics Committee of the Animal Experiment Center at the Hunan University of Chinese Medicine. The approval number was LLBH-202206070001, in compliance with animal use license SYXK (Xiang) 2019-0009. The pharmaceutical compound of the QEP consisted of Eucommia ulmoides, Psoralea corylifolia, walnut kernels, and garlic. These herbal constituents were sourced from the Chinese medicine pharmacy at the Second Affiliated Hospital of the Hunan University of Chinese Medicine. According to the “Pharmacopoeia of the People’s Republic of China” (2020 edition), the ingredients were mixed in a ratio of 16:8:5:4. After water decoction and concentration, 1 mL of the resultant solution contained 5.4 g of the herbal mixture.

#### 2.8.2 Major Reagents

The major reagents used in this study were the following: TRIzol (Invitrogen, Cat. No. 15596026); chloroform (Sinopharm Chemical Reagent Co., Ltd., Cat. No. 10008318); isopropanol (Sinopharm Chemical Reagent Co., Ltd., Cat. No. 80109218); anhydrous ethanol (Sinopharm Chemical Reagent Co., Ltd., Cat. No. 10009218); 50x TAE buffer (Beyotime Institute of Biotechnology, Cat. No. ST716); RNA agarose (Beyotime Institute of Biotechnology, Cat. No. ST004); radioimmunoprecipitation assay (RIPA) lysis buffer (Beyotime Institute of Biotechnology, Cat. No. P0013B); phosphatase inhibitor cocktail (Beyotime Institute of Biotechnology, Cat. No. P1081); phenylmethylsulfonyl fluoride (PMSF) solution (100 mM) (Biosharp, Cat. No. BL507A); Wnt5A, CTNNB1, IL-1β, MAPK14, MMP9, and MMP3 antibodies (Hunan Abcam Biotech Co., Ltd., Lot No. GR32031827); and Masson's trichrome, hematoxylin and eosin (H&E), and the safranin O/fast green staining kits (Hunan Abcam Biotech Co., Ltd., Lot Nos. GR32031828, GR32031829, and GR32031830).

#### 2.8.3 Animal Grouping, Drug Dosage, and Model Creation

After a 1-week acclimation period, the study was divided into two experimental phases. In the first phase, 12 rats were randomly selected and assigned to three groups, with four rats in each group: control, acupuncture (AC), and acupuncture with anhydrous ethanol (AC + ET). The AC group underwent the following model procedure: under abdominal anesthesia induced by sodium pentobarbital, a puncture was made with a 21 G needle at the intervertebral disc space between coccyx (Co) 6-7 and Co 7-8. The needle was inserted horizontally through the annulus fibrosus into the nucleus pulposus, rotated 360°, held for 30 sec, and then removed vertically, and the puncture site was compressed with a sterile cotton ball for 15 sec [26]. The AC + ET group additionally received a 5 μL injection of anhydrous ethanol into the nucleus pulposus following acupuncture [27]. Regarding blinding, we acknowledge that the need for oral gavage in our experiments limits the feasibility of implementing a fully blinded procedure. However, we took steps to maintain blinding in other aspects of the study. The remaining 30 rats were used in the second phase of the experiment, and they were assigned to five groups, with six rats in each group: control, model, and high (QH), medium (QM), and low (QL) doses of the QEP. Model construction for all groups, excluding the control group, replicated the acupuncture with anhydrous ethanol method to establish the IDD model. Beginning on the day after model establishment, the QEP dose groups received varying concentrations of the solution orally at 10.8, 5.4, and 2.7 g/kg once daily for 30 days. The control and model groups received an equivalent volume of saline in the same manner.

#### 2.8.4 Validation of the IDD Rat Model

In the model validation experiment, the control, AC, and AC + ET groups were euthanized using an anesthetic overdose 2 weeks after model establishment. Caudal vertebrae samples from segments Co 6-7 were collected for analysis. These tissues were immediately cryopreserved in liquid nitrogen for western blot (WB) and polymerase chain reaction (PCR) assessments, focusing on the apoptotic proteins caspase-3 and Bax in the nucleus pulposus. Furthermore, vertebrae from segments Co 7-8 were preserved in 10% formaldehyde, paraffin-embedded, sectioned according to standard protocols, and stained with H&E.

#### 2.8.5 Collection of Specimens from Animals used in the Second Phase of the Experiment

At the end of the solution orally, the rats were euthanized *via* anesthetic overdose. The soft tissues around the Co 6-7 intervertebral disc were carefully removed with forceps and a scalpel, revealing the vertebral body and annulus fibrosus. The boundary between the superior cartilaginous endplate and the vertebral body was carefully incised, allowing the endplate to be lifted and the gelatinous nucleus pulposus beneath to be visible. For the WB and PCR analyses, the nucleus pulposus was extracted using sterile micro-dissectors and immediately preserved in centrifuge tubes in liquid nitrogen. The caudal vertebrae at Co 8-9 were also dissected to obtain the Co 7-8 intervertebral disc. This disc was then fixed in a 4% paraformaldehyde solution and subjected to ethylenediaminetetraacetic acid decalcification. Thin paraffin sections of 4μm thickness were prepared for the subsequent Masson trichrome staining and safranin O-fast green staining.

#### 2.8.6 Western Blot

Protein concentrations in the intervertebral disc groups were quantified using the Coomassie Brilliant Blue assay. The samples were centrifuged at 12,000 rpm and 4ºC for 15 min, followed by the collection of the supernatant. For the electrophoretic analysis, 70 μg of protein per well was loaded onto a 10% sodium dodecyl sulfate-polyacrylamide gel. Initial electrophoresis was conducted at a constant voltage of 80 V for 30 min to ensure that the bromophenol blue dye front reached the separation gel and that the samples were aligned. The voltage was then increased to 120 V for 50-60 min to achieve full separation of the protein samples. The proteins were subsequently transferred to a polyvinylidene difluoride membrane and blocked with 5% skim milk at room temperature for 1 hour. The membrane was washed three times for 10 min each with tris-buffered saline and polysorbate 20 buffer (TBST), followed by overnight incubation with the primary antibody at 4ºC on a shaker. This was followed by three additional washes with TBST, and the membrane was incubated with the secondary antibody at room temperature for 1 hour. Then, it was washed three times for 10 min each with TBST. A chemiluminescence reagent was then applied to the membrane for detection. The bands were quantified using ImageJ software.

#### 2.8.7 Polymerase Chain Reaction

The total RNA was extracted from the intervertebral disc tissue using Trizol reagent and subsequently reverse-transcribed into cDNA. Quantitative real-time PCR (qRT-PCR) was performed using the SYBR Premix Ex Taq II kit, with GAPDH serving as the internal reference gene. Data analysis was conducted using the 2^−^ΔΔCT method. Primer details are presented in Table **2**.

### 2.9 Statistical Methods

Experimental data are presented as means ± standard deviations (x̄ ± s). Statistical analysis of the data was conducted using SPSS 11.5 software. Multiple group comparisons were made using a one-way analysis of variance followed by the Tukey-Kramer post hoc test. To control the false positive rate in multiple hypothesis tests, the Bonferroni correction method was applied. A *P*-value of less than 0.05 was defined as statistically significant.

## 3 RESULTS

### 3.1 Screening of Potential Active Ingredients and Targets of the QEP

**Fig. (1):**
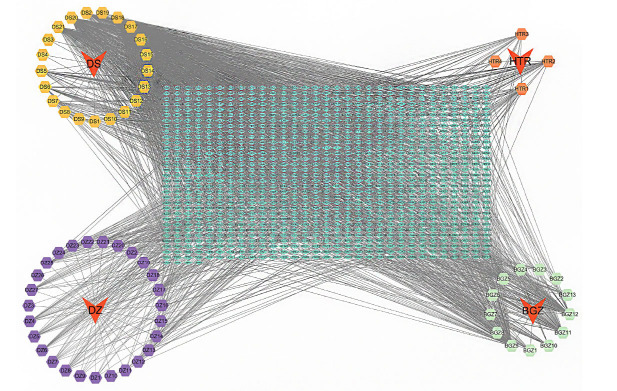
Predicted target network of the potential active components in the QEP.

Screening of potentially active components in the QEP using the TCMSP and BATMAN-TCM databases identified 65 potentially active ingredients in the pill: 27 from Eucommia ulmoides, 13 from Psoralea corylifolia, four from the walnut kernel, and 21 from garlic. The target information of these potential active components was subsequently processed and merged using the UniProt protein database, resulting in 1,093 targets. A “TCMs-Active Component-Target” network was constructed using Cytoscape 3.9.1 software (Fig. **1**) and analyzed using NetworkAnalyzer [28], with the results sorted by degree value. The analysis identified quercetin, berberine, isorhamnetin, and emodin as the top four potential active components that were likely responsible for the pharmacological effects of the QEP (Table **3**).

The red arrows around the periphery represent the four types of TCM in the QEP; the hexagons represent the potential active ingredients of each herb, with different colors indicating different potential active ingredients; and the green diamond in the middle represents the predicted targets.

### 3.2 Screening for Targets Associated with IDD and the “Liver/Kidney” Organ Targets

The GeneCards database provided a total of 2,108 targets associated with IDD. By searching the BioGPS database, we identified 812 and 307 genes that are specifically expressed in the liver and kidney, respectively. After merging and deduplicating these gene sets from both organs, a total of 1,113 unique genes were obtained.

### 3.3 PPI Network Analysis

**Fig. (2):**
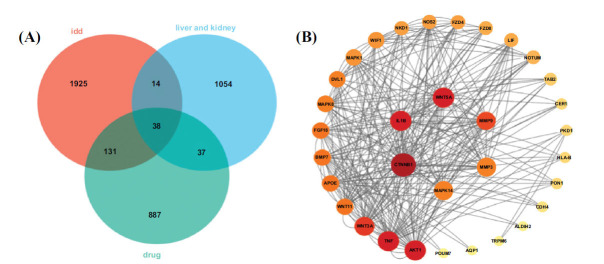
(**A**) Venn diagram showing the intersection targets of the “QEP-liver/kidney-IDD.” (**B**) Interaction network map of the 38 intersection genes.

After intersecting the QEP targets, IDD disease targets, and genes that are specifically expressed in the liver and kidney, we identified 38 overlapping targets associated with the “QEP-liver/kidney-IDD” (Fig. **2A**). These intersecting targets were then uploaded to the STRING database and imported into Cytoscape 3.9.1 for network optimization (Fig. **2B**). This network was composed of 33 nodes and 302 edges. By comparing the degree values of the nodes, we found that a higher degree value indicated a stronger correlation between the gene and other genes in the network [29].

### 3.4 KEGG and GO Enrichment Analyses

**Fig. (3):**
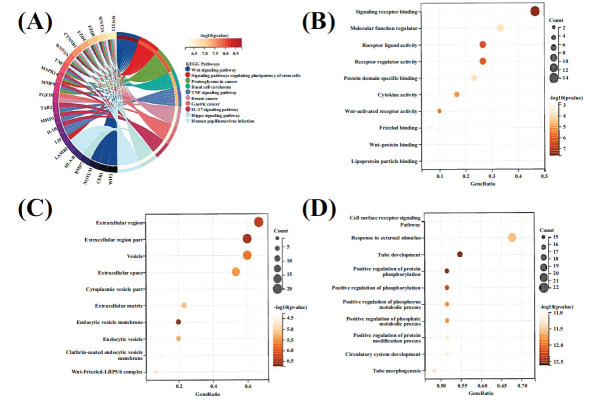
(**A**) KEGG pathway enrichment analysis of the QEP in the treatment of IDD. (**B**–**D**) GO function enrichment analysis of the QEP in the treatment of IDD (**B**: biological process; **C**: cellular component; and **D**: molecular function).

The GO term enrichment, including biological processes (BP), molecular functions (MF), and cellular components (CC), and KEGG pathway analyses were conducted on the 38 intersecting targets using a significance threshold of *P* < 0.05, with the results arranged in ascending order [30, 31]. The GO analysis revealed substantial enrichment in BPs, such as the cell surface receptor signaling pathways, response to external stimuli, tubular development, positive regulation of protein phosphorylation, phosphorus metabolic processes, and the development of the circulatory system and tubular morphology. In the CC category, enrichment was predominantly found in extracellular regions, vesicular parts, extracellular matrix, endocytic vesicle membranes, and the Wnt-Frizzled-LRP5/6 complex. Regarding the MF, notable enrichment was detected in signal receptor binding, molecular function regulator activity, receptor-ligand activity, protein domain-specific binding, cytokine activity, and Wnt-protein and lipoprotein particle binding (Fig. **3**).

### 3.5 Validation Set Verification

**Fig. (4):**
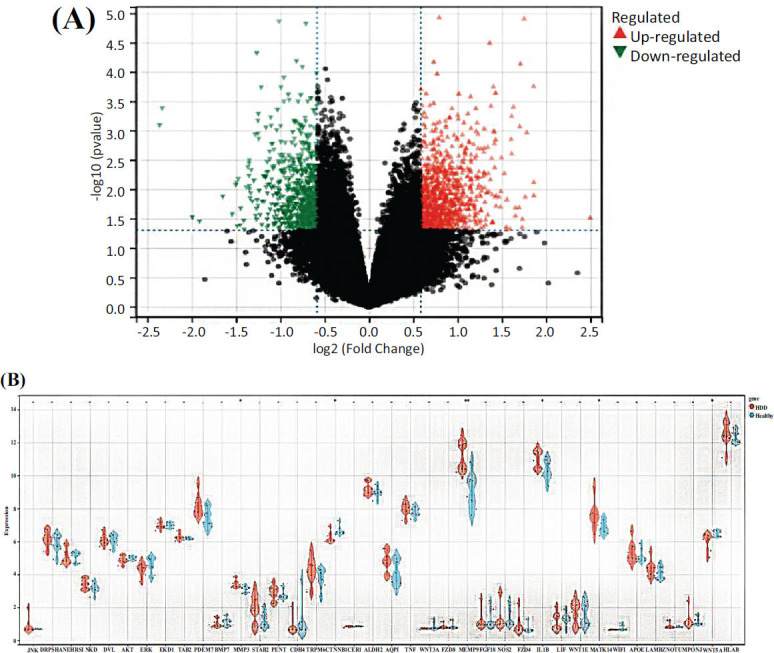
The validation set verified the difference in the intersection genes. (**A**) Volcano plot of the differentially expressed genes in the GSE124272 dataset. (**B**) Violin plot of the expression of the 38 intersection genes. *P < 0.05, **P < 0.01, and ***P < 0.001 compared with the healthy group.

A differential expression analysis was performed on the validation dataset, GSE124272, resulting in the identification of 1725 DEGs, with 930 upregulated and 795 downregulated DEGs (Fig. **4**). Subsequently, the 38 intersecting targets from the “QEP-Liver/Kidney-IDD” were integrated into the matrix to confirm whether the target set exhibited differential expression. The results revealed that 6 of the 38 intersecting genes displayed differential expressions: Wnt5A, CTNNB1, IL-1β, MAPK14, MMP9, and MMP3 (Fig. **4**).

### 3.6 Molecular Docking

The differentially expressed target genes (Wnt5A, CTNNB1, IL-1β, MAPK14, MMP9, and MMP3) and the top four potential active components of the QEP (quercetin, berberine, isorhamnetin, and emodin) were subjected to molecular docking interactions using AutoDock software for computational analysis. Fig. (**5**) shows the results as a heatmap of the binding energies. Binding energy, a vital indicator of the strength of molecular interactions, shows stronger binding as the value decreases [32]. In this study, all potential active components of the QEP exhibited binding energies of less than -4.0 kcal/mol. Among them, MMP9 and emodin had binding energies of -8.43 kcal/mol, while Wnt5A, CTNNB1, MAPK14, and IL-1β showed the strongest binding with berberine among all components, with energies of -7.72, -6.41, -8.2, and -7.17 kcal/mol, respectively. MMP3 showed the most potent binding activity with quercetin, with a binding energy of -7.46 kcal/mol. These findings demonstrate the significant binding activity between the potential active components of the QEP and the key target genes. To illustrate this binding more vividly, the docking results with the lowest binding energy for each key gene are visualized in Fig. (**6**).

### 3.7 Validation of the Rat IDD Model

**Fig. (7):**
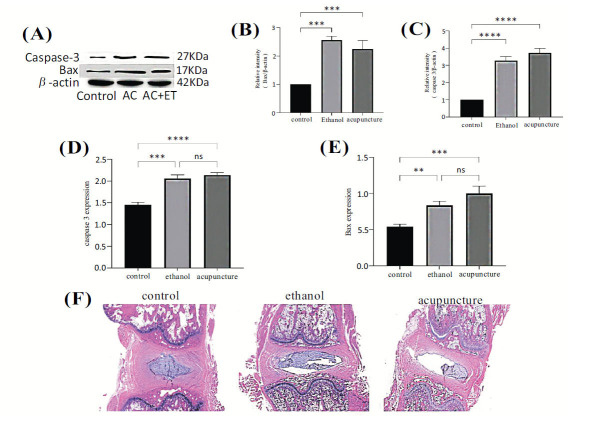
(**A**-**C**) Protein expression levels of caspase-3 and Bax in intervertebral disc tissues as determined using western blot assay. (**D** and **E**) The relative gene expression levels of caspase-3 and Bax in intervertebral disc tissues were determined using the polymerase chain reaction assay. (**F**) H&E staining images of intervertebral disc tissues from each group of rats (×100). Data are presented as the means ± SDs from triplicate experiments. *P < 0.05, **P < 0.01, and ***P < 0.001 compared with the blank group.

The WB analysis revealed a significant increase in caspase-3 expression in both the acupuncture and acupuncture plus anhydrous ethanol groups compared with the control group (*P* < 0.05). No significant difference in caspase-3 expression was observed between the two experimental groups (*P* > 0.05). Bax expression was significantly elevated in the acupuncture group compared with both the acupuncture plus anhydrous ethanol group and the control group (*P* < 0.05). There was no significant difference in Bax expression between the acupuncture plus anhydrous ethanol group and the control group. The qRT-PCR results demonstrated a significant increase in the mRNA levels of caspase-3 and Bax in both the AC group and the AC + ET group compared with the control group (*P* < 0.05). H&E staining revealed distinct morphological changes in the intervertebral disc tissues of the AC and AC + ET groups, as observed under a light microscope. These changes included partial annulus fibrosus rupture, abnormal nucleus pulposus structure, decreased nucleus pulposus cells, and indistinct or absent boundaries between the annulus fibrosus and nucleus pulposus compared with the control group. In conclusion, both the acupuncture method alone and the acupuncture plus anhydrous ethanol injection method were effective in creating a rat model of IDD, leading to apoptosis of nucleus pulposus cells (Fig. **7**).

### 3.8 Western Blot Analysis Results

**Fig. (8):**
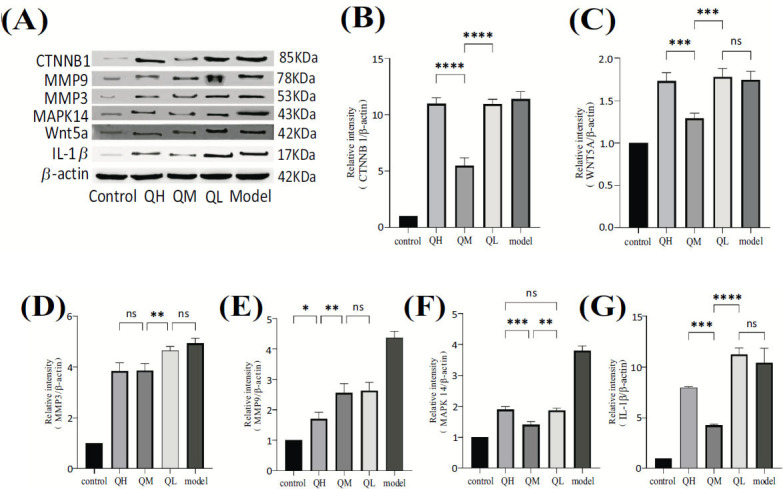
(**A**-**G**) Western blot assay results showing the protein expression levels of Wnt5A, CTNNB1, IL-1β, MAPK14, MMP9, and MMP3 in the blank, high-dose QEP, medium-dose QEP, low-dose QEP, and model groups. Data are presented as the means ± SDs from triplicate experiments. *P < 0.05, **P < 0.01, and ***P < 0.001 compared with the model group.

Compared with the control group, the model group showed significantly increased protein levels of Wnt5A, CTNNB1, IL-1β, MAPK14, MMP9, and MMP3 (*P* < 0.05). The high, medium, and low doses of the QEP significantly reduced the protein levels of CTNNB1, Wnt5A, and IL-1β in intervertebral disc tissues compared with the model group (*P* < 0.05). The low-dose QEP group did not show significant differences in the MAPK14, MMP9, and MMP3 levels compared with the model group (*P* > 0.05). Additionally, the high-dose QEP group exhibited significantly lower levels of CTNNB1 and MMP9 than the medium- and low-dose groups (*P* < 0.05). There were no significant differences in the MAPK14 and MMP3 levels between the high- and medium-dose groups (*P* > 0.05). Furthermore, the low-dose QEP group had significantly lower Wnt5A levels than the high- and medium-dose groups (*P* < 0.05) (Fig. **8**).

### 3.9 qRT-PCR Analysis Results

**Fig. (9):**
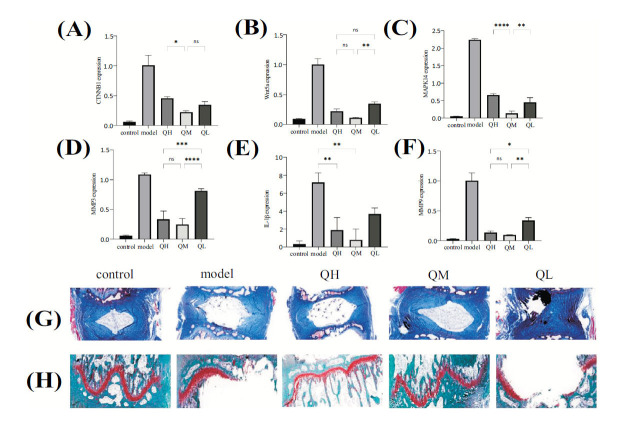
(**A**-**F**) Relative gene expression levels of Wnt5A, CTNNB1, IL-1β, MAPK14, MMP9, and MMP3 in intervertebral disc tissues from each rat group; (**G**) Masson's trichrome staining (×200); and (**H**) Safranin O-fast green staining (×400). Data are presented as the means ± SDs from triplicate experiments. *P < 0.05, **P < 0.01, and ***P < 0.001 compared with the model group.

Compared with the control group, the model group exhibited a significant increase in the mRNA expression levels of Wnt5A, CTNNB1, IL-1β, MAPK14, MMP9, and MMP3 (*P* < 0.05). In contrast, the low-, medium-, and high-dose QEP groups showed significant decreases in the mRNA expression of these markers in intervertebral disc tissues compared with the model group (*P* < 0.05). Further comparison revealed that compared with the high-dose QEP group, significant reductions in the mRNA expression levels of CTNNB1, IL-1β, and MAPK14 were observed in the medium-dose QEP group (*P* < 0.05). In comparison with the low-dose QEP group, the medium-dose QEP group exhibited significantly decreased mRNA expression levels of Wnt5A, IL-1β, MAPK14, MMP9, and MMP3 (*P* < 0.05). However, while there were significant differences in the mRNA expression levels of Wnt5A, IL-1β, MMP9, and MMP3 between the high-dose and medium-dose QEP groups (*P* < 0.05), no significant differences were observed for CTNNB1 and MAPK14 (*P* > 0.05) (Fig. **9A**-**F**).

### 3.10 Masson's Trichrome and Safranin O-fast Green Staining Results

After Masson's trichrome staining, the intervertebral disc tissues exhibited the following characteristics: collagen fibers appeared blue, nucleus pulposus cells were deep red or brown, the cytoplasm was red, and a distinct demarcation existed between the nucleus pulposus tissue and fibrous annulus tissue. After model induction, fissures formed with in the inner layer of the fibrous annulus, blurring the boundary between the nucleus pulposus and the fibrous annulus, leading to instances of nucleus pulposus disappearance. Following intervention with the QEP, a significant increase in the collagen fiber content in the disc tissues was observed, and the fissures in the nucleus pulposus and fibrous annulus tissues were reduced. Notably, the disc morphology of the medium-dose QEP group was superior to that of the high- and low-dose QEP groups (Fig. **9G**).

After safranin, O-fast green staining, the cartilage endplates of the model group exhibited defects in integrity and disorganized layering compared with the control group, with lighter safranin O staining intensity. After intervention with the TCM QEP by gavage, the structural morphologies of the cartilage endplates in the high-, medium-, and low-dose QEP groups were more intact compared with the model group, with deeper and more uniform safranin O staining and a marked decrease in the number of hypertrophic degenerative vacuolated cells (Fig. **9H**).

## 4 DISCUSSION

Using network pharmacology, in this study, we identified quercetin, berberine, isorhamnetin, and emodin as key active components of the QEP. Validation of the 38 intersecting targets in the “Qing’e Pill-Liver/Kidney-IDD” network revealed six candidates with differential expressions compared with healthy controls: Wnt5A, CTNNB1, IL-1β, MAPK14, MMP9, and MMP3. The GO and KEGG analyses indicated that these targets are involved in Wnt receptor activation, Frizzled binding, Wnt-protein interactions, and the TNF signaling pathways. The Wnt signaling pathway significantly affects intervertebral disc cell proliferation, senescence, apoptosis, and extracellular matrix changes [33]. Activation of the Wnt signaling pathway with 20 mmol/L LiCl reduces nucleus pulposus cell numbers, arrests the cell cycle, and slows cell proliferation [34]. Additionally, high POSTN expression in severely degenerated discs induces apoptosis in nucleus pulposus cells, while Wnt signaling inhibition reduces this effect [35]. Specifically, Wnt5A, a crucial regulator within the Wnt pathway, induces apoptosis and extracellular matrix degradation through the non-canonical pathway [36]. Overactivation of CTNNB1 in the canonical pathway results in abnormal cell proliferation and alterations to the extracellular matrix [37]. In addition, factors such as IL-1β [38], MAPK14 [39], MMP9 [40], and MMP3 [41] can contribute to inflammation and matrix degradation, potentially further interacting with Wnt signaling to exacerbate disc degeneration. These results suggest that inhibiting Wnt signaling may reverse POSTN-induced apoptosis, offering a potential strategy for improving IDD. The literature research showed that the primary active components of the QEP, berberine, and isorhamnetin, have significant antioxidant and anti-inflammatory properties that are crucial for maintaining intervertebral disc stability and health [42, 43]. Specifically, berberine regulates the Bcl-2/Bax ratio, enhances apoptotic signaling, and increases apoptosis-inducing factors, promoting cell apoptosis in degenerative disc regions [44]. Isorhamnetin, an effective free radical scavenger with antioxidant activity, reduces oxidative stress and protects disc cells [45]. Regarding interactions with the Wnt pathway, berberine inhibits Wnt signaling, lowers caspase-catenin levels, and reduces apoptosis [46]. Isorhamnetin may influence signal transduction and cellular responses by modulating key Wnt pathway proteins, such as Dishevelled, GSK-3β, and β-catenin [47].

We used both molecular docking and animal experiments to validate the network pharmacology analysis results. In this study, all potential active components of the QEP exhibited binding energies below −4.0 kcal/mol, indicating strong binding affinity with key target genes. The results highlight that the active compounds collectively target key inflammatory factors and matrix-degrading enzymes, thereby exerting anti-inflammatory, antioxidant, and matrix-preserving effects to mitigate the progression of intervertebral disc degeneration [48-51]. In animal experiments, the high-, medium-, and low-dose QEP groups significantly reduced the expression levels of Wnt5A, CTNNB1, IL-1β, MAPK14, MMP9, and MMP3 compared with the model group. This indicates that the QEP can inhibit pathway activation and inflammatory factor release by targeting the Wnt, MAPK, and MMP pathways. Among the three dosage groups, the medium-dose QEP demonstrated the best therapeutic effect, supporting the network pharmacology results. Wnt5A and CTNNB1 are crucial in IDD. Wnt5A, a key regulator in the Wnt signaling pathway, promotes apoptosis and extracellular matrix degradation by activating the non-canonical Wnt pathway [52]. CTNNB1, involved in the canonical Wnt pathway, can cause dysregulated cell proliferation and changes in extracellular matrix components when overactivated [53]. MAPK14, a significant member of the MAPK family, controls inflammatory responses. IL-1β and TNF-α activate the p38 MAPK pathway, increasing the MMP3/TIMP-1 ratio, disrupting the extracellular matrix in nucleus pulposus cells, and accelerating disc degeneration [53]. MMP3, MMP9, and matrix metalloproteinases contribute to extracellular matrix degradation, leading to imbalances and the onset of IDD [54, 55]. IL-1β, an important inflammatory factor in disc degeneration, induces excessive apoptosis of disc cells, and this exacerbates the activation of the Wnt, MAPK, and MMP pathways, further advancing disc degeneration [56]. The combined effect of the Wnt signaling, MAPK, and MMP pathways activates MAPK14, increases the levels of inflammatory factors such as IL-1β, and leads to an overexpression of MMP9 and MMP3, accelerating degeneration [57]. In summary, the QEP improves IDD by modulating targets such as Wnt5A, CTNNB1, IL-1β, MAPK14, MMP9, and MMP3 and by regulating the Wnt signaling and MAPK/MMP pathways. These findings are crucial for understanding IDD mechanisms and the therapeutic potential of the QEP.

However, this study has several limitations. First, the analysis was based on validated components, targets, and pathways, and this may have resulted in the omission of some unrecognized components or targets. Second, the interactions among the Wnt pathway, MAPK pathway, and MMPs were not examined. While molecular docking results showed potential binding activities, the complexity of *in vivo* physiological environments may limit their applicability. Furthermore, the relatively small sample size in the animal experiments may restrict the generalizability of the findings. Future research will involve additional *in vivo* cellular experiments to explore the interactions among these factors and pathways in greater detail, alongside a larger sample size and clinical data to enhance the translational relevance of the findings.

## CONCLUSION

This study posited that the QEP mitigated IDD by inhibiting the Wnt/MAPK/MMP signaling pathways, thereby reducing the release and activation of related factors, subsequently diminishing cell apoptosis and extracellular matrix degradation. This research provides preliminary evidence supporting the potential of TCM components for the treatment of IDD.

## Figures and Tables

**Fig. 5:**
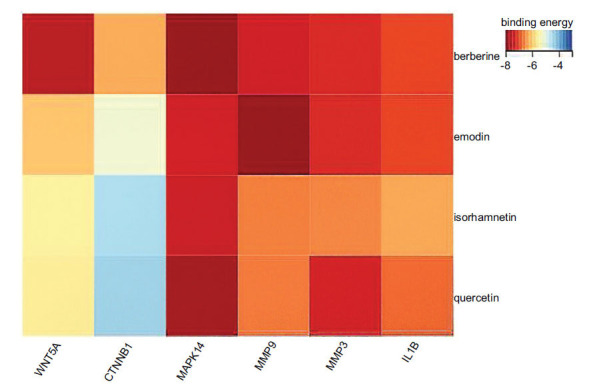
Heatmap of the binding energy for molecular docking of the top four active components in the QEP (quercetin, berberine, isorhamnetin, and emodin) with differentially expressed target genes (Wnt5A, CTNNB1, IL-1β, MAPK14, MMP9, and MMP3).

**Fig. 6:**
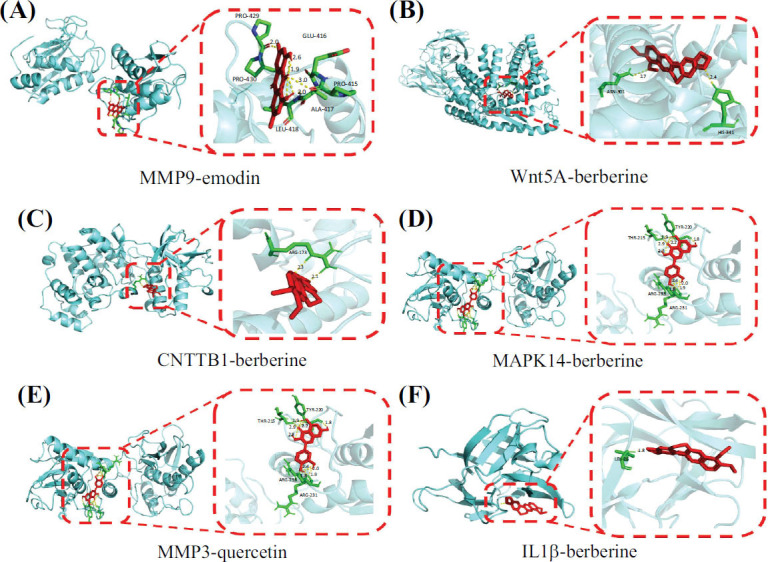
(**A**-**F**) Three-dimensional virtual docking diagrams of Wnt5A, CTNNB1, MAPK14, and IL-1β with berberine; MMP9 with emodin; and MMP3 with quercetin.

**Table 1:** Software and databases used in this study.

**S. No.**	**Database/Software**	**Webpage**
1	TCMSP	https://tcmspw.com/tcmsp.php
2	BATMAN-TCM	http://bionet.ncpsb.org.cn/batman-tcm/
3	STRING	https://string-db.org/
4	GeneCards	https://www.genecards.org/
5	UniProt	https://www.uniprot.org/
6	Protein Data Bank	https://www.rcsb.org
7	BioGPS	http://biogps.org/
8	Gene Expression Omnibus	https://www.ncbi.nlm.nih.gov/geo/
9	Cytoscape 3.7.2	https://cytoscape.org/
10	AutoDockTools 1.5.6	https://autodocksuite.scripps.edu/adt/
11	AutoDock Vina 1.1.2	https://vina.scripps.edu/
12	R 4.2.3	https://www.r-project.org/
13	ImageJ	https://imagej.net/software/imagej/
14	SPSS 11.5	https://www.ibm.com/cn-zh/spss

**Table 2:** Primer sequences.

**Gene**	**Forward (5'-3')**	**Reverse (5'- 3')**
GAPDH	ACTCTACCCACGGCAAGTTC	TGGGTTTCCCGTTGATGACC
Bax	ATCGAGCAGAGAGGATGGCT	ACTCGCTCAGCTTCTTGGTG
caspase-3	GGCTGGAACCCTTGTTTTGG	TTCGCACACGGTTTTCCTCT
IL-1β	ACTATGGCAACTGTCCCTGAAC	GTGCTTGGGTCCTCATCCTG
wnt5a	AGTCCTGCTTTGAATCGTCCC	ATTACAACCTGGGCGAAGGAG
β-catenin	ATGACGTAGAAACAGCCCGT	AGCGTGGTGATGGCGTAGAA
MAPK14	GCCCGAGCGATACCAGAACC	GCTTCTTCACTGCCACACGAT
MMP3	CCTCTGAGTCTTTTCATGGAGGG	ACTTGAGGTTGACTGGTGCC
MMP9	ATGGGAGAGAAGCAGTCCCT	GGCCTTTAGTGTCTCGCTGT

**Table 3:** Top 10 potential active ingredients of the QEP by degree value.

**Molecule Name**	**Degree**	**Medicine**
Berberine	399	Garlic
Isorhamnetin	189	Eucommia
Emodin	120	Psoralen
Quercetin	108	Garlic
Divinyl sulfide	99	Walnut
N-Methylmescaline	97	Garlic
Backuchiol	75	Psoralen
Beta-sitosterol	58	Eucommia
Xanthotoxin	58	Psoralen
Sulfurenic acid	48	Garlic

## Data Availability

All data generated or analyzed during this study are included in this published article.

## LIST OF ABBREVIATIONS

BATMAN-TCM: Bioinformatics Analysis Tool for Molecular Mechanisms of Traditional Chinese Medicine
DAVID: Database for Annotation, Visualization, and Integrated Discovery
DEGs: Differentially Expressed Genes
GO: Gene Ontology
IDD: Intervertebral Disc Degeneration
KEGG: Kyoto Encyclopedia of Genes and Genomes
PDB: Protein Data Bank
QEP: Qing’e Pill
TCM: Traditional Chinese Medicine
TCMSP: Traditional Chinese Medicine Systems Pharmacology Database and Analysis Platform
